# Burden, risk factors, and projections of ischemic heart disease in China (1990–2021): findings from the 2021 GBD study

**DOI:** 10.3389/fcvm.2025.1549147

**Published:** 2025-02-26

**Authors:** Sikai Xu, Zhiyang Liu, Mu Tang, Chunli Xu

**Affiliations:** ^1^ Department of Medical Genetics, Jiangxi Medical College, The Second Affiliated Hospital, Nanchang University, Nanchang, Jiangxi, China; ^2^ Department of Breast Surgery, Jiangxi Cancer Hospital, Nanchang, Jiangxi, China; ^3^ Department of Anesthesiology, Tongji Medical College, Union Hospital, Huazhong University of Science and Technology, Wuhan, China

**Keywords:** China disease burden, ischemic heart disease, decomposition analysis, joinpoint regression, risk factors, prediction

## Abstract

**Background:**

Ischemic heart disease (IHD) remains a major public health challenge in China. This study aimed to comprehensively analyze the burden of IHD, its risk factors, and future trends from 1990 to 2021 using the Global Burden of Disease database.

**Methods:**

We assessed IHD trends in incidence, prevalence, mortality, and disability-adjusted life years (DALYs) stratified by age (greater than 15 years) and gender. Age-standardized rates, average annual percentage changes, and joinpoint regression analyses were used to evaluate temporal trends. Decomposition and frontier analyses were conducted to identify key contributors to the IHD burden, while future projections were generated for the next 15 years.

**Results:**

In 2021, the number of IHD incident cases, prevalent cases, deaths, and DALYs in China were 3.17, 3.25, 3.57, and 2.62 times higher than those in 1990, respectively. Age-standardized mortality rates and age-standardized DALYs rates demonstrated an initial increase, followed by a gradual decline. Males showed higher IHD burden during middle age, while elderly females surpassed males in the later years. Aging, high systolic blood pressure, ambient particulate matter pollution, elevated low-density lipoprotein cholesterol, and smoking were the primary drivers of IHD burden. Future projections suggest a declining incidence and prevalence among males but increasing trends in females, with DALYs expected to rise significantly in the female population.

**Conclusions:**

The burden of IHD in China has evolved significantly over the past three decades, driven by demographic and environmental factors. While prevalence and incidence have risen, mortality and DALYs have shown a recent decline, reflecting shifts in disease patterns. Age and gender disparities are evident, with middle-aged males and elderly females disproportionately affected. Key contributors, such as high blood pressure and pollution, highlight the need for targeted interventions. Gender-specific public health strategies, alongside improved environmental and health policies, are essential to mitigating the future burden of IHD in China.

## Introduction

Ischemic heart disease (IHD), commonly referred to as coronary artery disease, is caused by reduced blood flow to the heart muscle due to narrowed or blocked coronary arteries (1). Clinically, it manifests as chest pain (angina), myocardial infarction, or sudden cardiac death, posing a significant threat to global health (1, 2). As one of the leading causes of death and disability worldwide, IHD imposes substantial health and economic burdens (3). In 2021, there were over 19.16 million heart failure (HF) cases attributed to IHD globally, with an age-standardized prevalence rate (ASPR) of 228.31 per 100,000 and an age-standardized years lived with disability (ASYLD) rate of 20.43 per 100,000. Since 1990, ASPR and ASYLD have increased by 2.87%, primarily due to population growth and aging (4). By 2050, the global burden of IHD is projected to rise sharply, with incidence, prevalence, deaths, and disability-adjusted life years (DALYs) increasing by 116%, 106%, 80%, and 62%, respectively, compared to 2021. Regions with lower sociodemographic index (SDI) scores face disproportionately higher burdens, and the disease remains more prevalent among men and older adults (5).

The prevalence and burden of IHD vary significantly across regions and populations due to differences in socioeconomic factors, lifestyle, and healthcare systems (6, 7). Globally, low- and middle-income countries, including China, are experiencing an increasing incidence and mortality rate of IHD driven by rapid urbanization, aging populations, and lifestyle changes (8, 9). Notably, the epidemiology of cardiovascular disease (CVD) also shows substantial variation between countries. According to recent data, CVD remains the leading cause of death in China, with about 106 million CVD patients, including 33 million with stroke. In 2019, IHD was the second leading cause of cardiovascular mortality, with an estimated mortality rate of 147.3 per 100,000 and an age-standardized mortality rate (ASMR) of 142.1 per 100,000. Stroke and IHD combined accounted for over 87% of all CVD deaths (10).

Between 1990 and 2019, the burden of IHD in China steadily increased, with premature mortality due to CVD significantly exceeding the global average and surpassing that of many high-income countries (11). Cardiovascular disease accounts for approximately 40% of all deaths in China, with IHD as a major contributor to this statistic (12). The top five risk factors contributing to this burden are elevated systolic blood pressure, high low-density lipoprotein cholesterol (LDL-C), smoking, ambient particulate matter pollution, and high sodium intake (13).

This study aims to provide valuable insights into the trends of IHD in China by analyzing the most recent data from 1990 to 2021. Using Joinpoint regression and Age-Period-Cohort (APC) models, the study evaluates the trends in incidence, prevalence, mortality, and DALYs. Additionally, frontier analyses are employed to explore the 32-year development trends across 204 countries, identifying potential learning exemplars. The study also investigates key risk factors and projects future trends, contributing crucial perspectives for mitigating the disease burden of IHD.

## Materials and methods

### Data sources

The Global Burden of Disease (GBD) database, developed by the Institute for Health Metrics and Evaluation (IHME), is a comprehensive global research initiative that quantifies health outcomes and risk factors across countries and regions. By synthesizing data from epidemiological surveys, registries, administrative records, and systematic reviews, the GBD provides estimates of the burden of diseases, injuries, and risk factors using standardized metrics such as incidence, prevalence, mortality, years of life lost (YLLs), years lived with disability (YLDs), and DALYs. This study utilized data from the latest GBD 2021 database, focusing on IHD in China (Mainland China, excluding Taiwan Province) as the research subject. Data on incidence, prevalence, mortality, and DALYs associated with IHD for individuals aged over 15 years were extracted to comprehensively evaluate its disease burden. Additionally, risk factor data associated with IHD were obtained for further analysis. Since the GBD database does not involve direct participation of human subjects, ethical approval was not required for this study.

### Statistical analysis

The Joinpoint regression model is a statistical technique widely utilized for analyzing trends in time-series data, particularly in the fields of epidemiology and public health. This method is particularly effective in identifying changes in trends over time by segmenting the data into distinct intervals, or “joinpoints” where the slope of the trend may change (14). In this study, we utilized Joinpoint software version 5.1.0.0 to analyze trends in the burden of disease over the period from 1990 to 2021. The model was configured with a maximum of six joinpoints to identify the optimal temporal trends. Additionally, the software was employed to calculate the Average Annual Percentage Change (AAPC) as well as the Annual Percentage Change (APC) for each identified segment, providing a detailed characterization of trend dynamics across the study period.

The APC model is a statistical framework extensively applied in epidemiology and social sciences to investigate the independent and combined effects of age, time period, and birth cohort on various health outcomes and demographic trends. By disentangling the contributions of these three dimensions, the APC model facilitates a deeper understanding of how age-related biological changes, period-specific environmental or societal factors, and generational influences interact to shape disease incidence, mortality, and other health indicators over time. In this study, we employed a web tool to examine the effects of these dimensions on the burden of disease (15). Data spanning a 30-year period from 1992 to 2021 were analyzed, with each time period divided into 5-year intervals. This approach enabled us to quantify and interpret the distinct and overlapping contributions of age, period, and cohort factors to temporal trends in disease burden.

The decomposition analysis is an effective tool for breaking down overall differences between two time points or two populations into multiple contributing components, thereby revealing the underlying factors responsible for observed changes. In this study, decomposition analysis was employed to attribute the changes in disease burden to aging, population growth, and epidemiological shifts, aiming to explore the driving forces behind the changes in disease burden from 1990 to 2021.

Additionally, a cluster analysis was conducted on the 27 risk factors associated with IHD, focusing on their contributions to disease burden in terms of mortality and DALYs. This approach allowed us to identify both the risk factors currently contributing the most to the burden of IHD and those with the greatest change in burden over the past 32 years.

Frontier analysis in this study was conducted using the Data Envelopment Analysis (DEA) approach, which is a linear programming-based method. DEA identifies optimal performance by constructing a “frontier boundary” encompassing all decision-making units, with units below the frontier considered inefficient. To evaluate the robustness of the DEA, we applied a Bootstrap procedure with 100 iterations, generating multiple resampled datasets to assess stability. The final results were visualized using Locally Weighted Regression (LOESS) to illustrate the trends and performance of DALYs attributed to IHD in China, compared to 204 countries, over the period from 1990 to 2021.

An Autoregressive Integrated Moving Average (ARIMA) model was employed to forecast changes in the burden of IHD in China over the next 15 years. The auto.arima function was used to automatically fit the ARIMA model, determining the optimal values for (the order of the autoregressive model), *d* (the degree of differencing), and (the order of the moving-average model). Various combinations of *p*, *d*, and *q* were tested, and the best-fitting model was selected based on the Akaike Information Criterion (AIC) or Bayesian Information Criterion (BIC), ensuring an accurate and robust prediction of future disease burden trends. All statistical analyses were performed using R software version 4.3.2, and *P* < 0.05 was considered statistically significant.

## Result

### Burden of IHD in China

**Figure 1:**
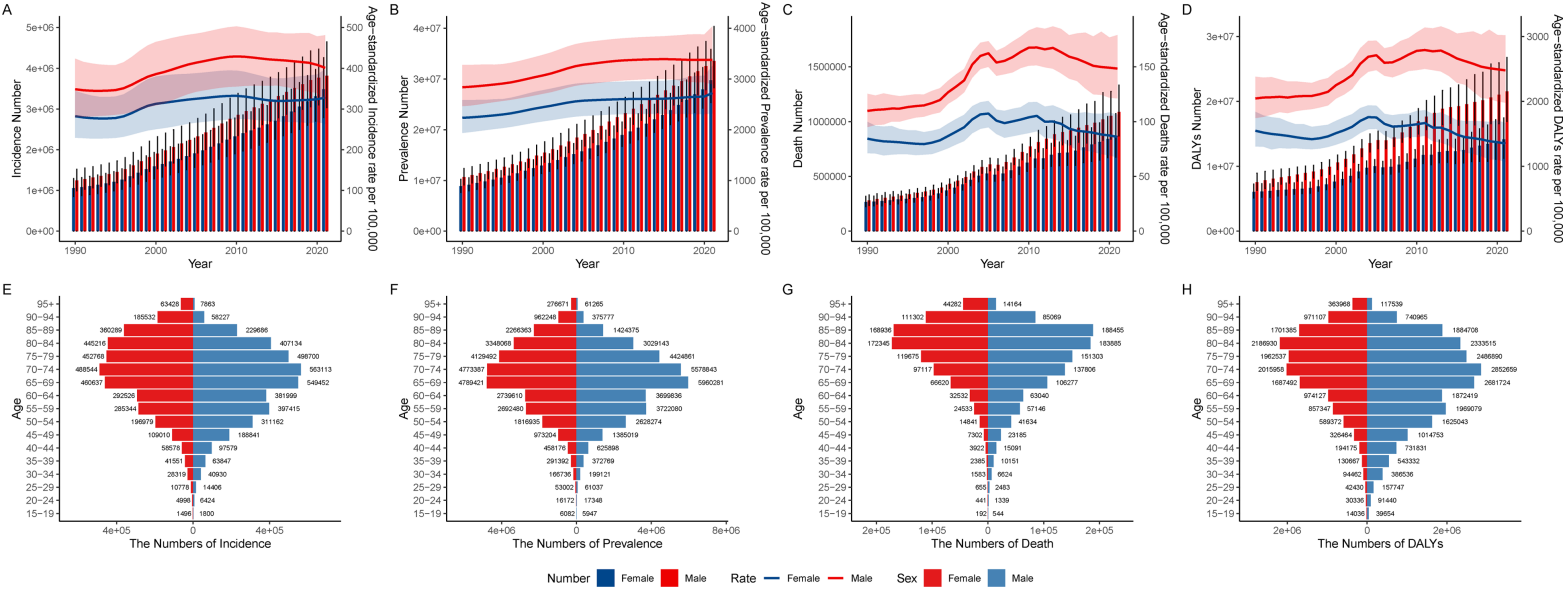
Trends in disease burden counts and age-standardized rates of IHD in males and females in China, 1990–2021: **(A)** incidence, **(B)** prevalence, **(C)** mortality, **(D)** DALYs. The age-specific counts of **(E)** incidence, **(F)** prevalence, **(G)** mortality, and **(H)** DALYs in males and females in China in 2021.

The burden of IHD in China, measured in terms of case numbers, has exhibited a continuous increase from 1990 to 2021, encompassing prevalence, incidence, mortality, and DALYs, with males generally outnumbering females across these metrics. However, in the past decade, the ASMR and age-standardized disability-adjusted life years rate (ASDR) have shown a gradual decline in both males and females, alongside a downward trend in the age-standardized incidence rate (ASIR) among males (Figures 1A–D). The total number of incident cases rose from 2,301,644 (95% UI: 18,61,969–2,792,193) in 1990 to 7,304,573 (95% UI: 5,815,313–8,949,995) in 2021 (Table 1), with the disease onset beginning at age 15 and an exponential increase in cases observed in the 40 to 69 age group. While the number of incident cases is higher in males than in females from ages 15 to 79, females surpass males in terms of incident cases starting at age 80 (Figure 1E).

**Table 1:** The number and age-standardized rates of incidence, prevalence, mortality, and DALYs for IHD in 1990 and 2021, along with the AAPC from 1990 to 2021.

Characteristics	1990		2021		1990–2021
Incidence	No. (95% UI)	ASIR (95% UI)	No. (95% UI)	ASIR (95% UI)	AAPC (95% CI)
Both	23,01,644 (1861969,2792193)	315.31 (255.53,382.49)	73,04,573 (5815313,8949995)	365.67 (293.32,440.07)	0.49 (0.43,0.54)
Male	12,44,366 (991819,1520020)	348.59 (279.98,423.58)	38,18,580 (3034881,4650552)	401.19 (321.97,481.19)	0.46 (0.41,0.52)
Female	10,57,277 (852792,1292714)	282.24 (229.4,344.51)	3,485,993 (2792003,4259778)	328.08 (263.97,397.25)	0.47 (0.36,0.57)
Prevalence	No. (95% UI)	ASPR (95% UI)	No. (95% UI)	ASPR (95% UI)	AAPC (95% CI)
Both	19,50,5463 (16754811,22537174)	2526.44 (2189.97,2914.97)	63,33,1312 (53812324,76196537)	3042.35 (2601.68,3629.87)	0.60 (0.58,0.62)
Male	10,64,8703 (9142880,12348341)	2838.18 (2466.64,3277.45)	33,57,1872 (28288613,40380503)	3379.15 (2895.87,4036.35)	0.56 (0.53,0.59)
Female	88,56,760 (7651263,10222044)	2235.32 (1938.26,2585.03)	29,75,9440 (25359422,35857849)	2724.16 (2320.22,3259.05)	0.63 (0.58,0.67)
Deaths	No. (95% UI)	ASMR (95% UI)	No. (95% UI)	ASMR (95% UI)	AAPC (95% CI)
Both	547,845 (486106,617006)	94.14 (84.01,105.89)	19,56,859 (1634478,2280131)	110.91 (92.42,128.56)	0.49 (0.23,0.75)
Male	280,999 (235025,331060)	109.77 (95.03,125.37)	10,88,197 (868768,1332608)	148.4 (121.2,178.97)	0.95 (0.60,1.31)
Female	266,846 (223803,318705)	84.41 (71.18,99.75)	868,663 (676759,1073178)	86.1 (67.1,106.39)	0.03 (−0.24,0.29)
DALYs	No. (95% UI)	ASDR (95% UI)	No. (95% UI)	ASDR (95% UI)	AAPC (95% CI)
Both	13,62,4112 (12056606,15466092)	1771.13 (1574.76,1990.67)	35,67,2627 (29920273,41738946)	1856.51 (1548.73,2159.82)	0.11 (−0.09,0.32)
Male	75,56,608 (6300651,8936138)	2044.54 (1742.46,2379.74)	21,52,9834 (16894342,26751533)	2477.99 (1979.87,3018.01)	0.60 (0.36,0.84)
Female	60,67,504 (5114360,7253345)	1548.06 (1305.17,1839.24)	14,14,2793 (11236414,17447300)	1350.62 (1068.58,1666.75)	−0.48 (−0.71,−0.24)

Similarly, the total number of prevalent cases grew substantially, increasing from 19,505,463 (95% UI: 16,754,811–22,537,174) in 1990 to 63,331,312 (95% UI: 53,812,324–76,196,537) in 2021 (Table 1). Among individuals aged 80 and above, the number of prevalent cases among females exceeded that of males (Figure 1F). Regarding mortality, the number of deaths due to IHD escalated from 547,845 (95% UI: 486,106–617,006) in 1990 to 1,956,859 (95% UI: 1,634,478–2,280,131) in 2021 (Table 1). Before the age of 70, males consistently experienced higher death counts across all age categories, but from age 85 onward, the mortality count among females exceeded that of males (Figure 1G). Lastly, the DALYs attributed to IHD increased from 13,624,112 (95% UI: 12,056,606–15,466,092) in 1990 to 35,672,627 (95% UI: 29,920,273–41,738,946) in 2021 (Table 1). Notably, males had significantly higher DALYs before the age of 65, but women surpassed men in this metric after age 90 (Figure 1H).

From 1990 to 2021, the ASIR and ASPR of IHD in both males and females in China showed an overall upward trend. The AAPC for ASIR was 0.46 (95% CI: 0.41, 0.52) in males and 0.47 (95% CI: 0.36, 0.57) in females. Similarly, the AAPC for ASPR was 0.56 (95% CI: 0.53, 0.59) in males and 0.63 (95% CI: 0.58, 0.67) in females. Moreover, the ASMR in males exhibited an increasing trend with an AAPC of 0.95 (95% CI: 0.60, 1.31), as did the ASDR with an AAPC of 0.60 (95% CI: 0.36, 0.84). In contrast, the ASMR in females remained relatively stable (AAPC = 0.03, 95% CI: −0.24, 0.29), while the ASDR in females showed a decreasing trend (AAPC = −0.48, 95% CI: −0.71, −0.24).

### Joinpoint regression analysis of the burden of IHD in China

**Figure 2:**
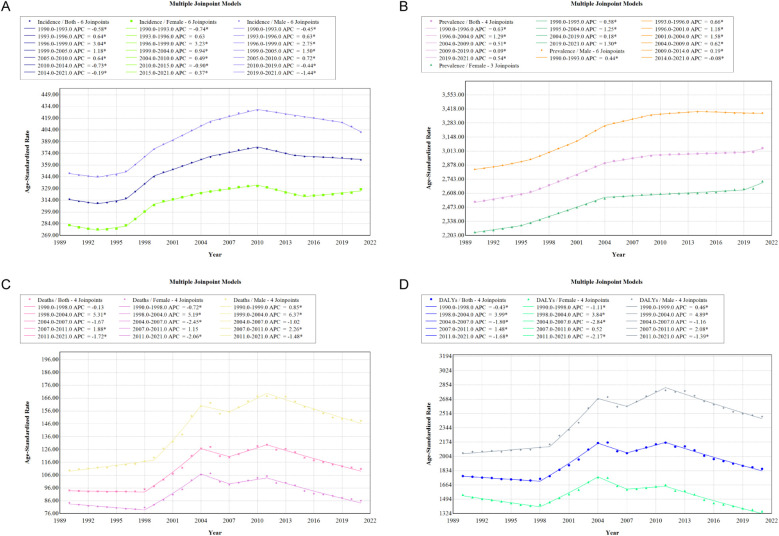
APC in ASIR **(A)**, ASPR **(B)**, ASMR **(C)**, and ASDR **(D)** for IHD in males and females in China, 1990–2021 (* indicates *P*-value < 0.05 and statistically significant).

To visually represent the trends and inflection points of IHD in China from 1990 to 2021, we conducted a joinpoint regression analysis (Figures 2A–D). The results indicate that for both males and females, there was a significant increase in the ASIR of IHD between 1996 and 1999, with an APC of 3.04 (*P* < 0.05). Following this period, the incidence rate exhibited a gradual increase until 2010, when it began to decline (2010–2014 APC = −0.73, *P* < 0.05). Notably, from 2014 onward, the ASIR for females continued to rise (APC = 0.37, *P* < 0.05), while the ASIR for males continued to decline (APC = −1.44, *P* < 0.05).

From 1990 to 2021, the ASPR of IHD in China primarily experienced rapid growth between 1996 and 2004 (APC = 1.29, *P* < 0.05). Notably, the annual percentage change in the ASPR for females from 2019 to 2021 (APC = 1.30, *P* < 0.05) was significantly higher than that for males (APC = −0.08).

During the period from 1990 to 2021, the ASMR exhibited a significant increase primarily between 1998 and 2004 (APC = 5.31, *P* < 0.05). However, since 2011, the ASMR has shown a declining trend (APC = −1.72, *P* < 0.05). Similarly, the ASDR exhibited a comparable trend, with an annual percentage change of 3.99 between 1998 and 2004 (*P* < 0.05), followed by a year-on-year decrease after 2011 (APC = −1.68, *P* < 0.05).

### APC model analysis

**Figure 3:**
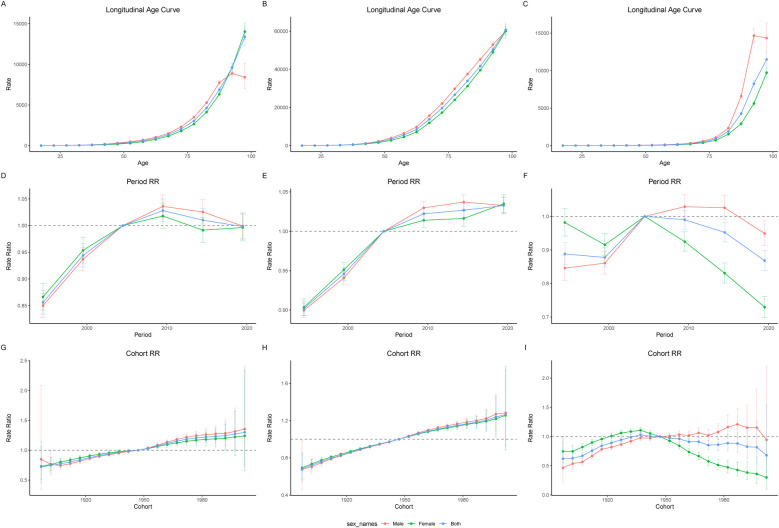
Longitudinal age curves of IHD incidence **(A)**, prevalence **(B)**, and mortality **(C)** in males and females from 1992 to 2021. Panels **(D)**, **(E)**, and **(F)** represent the Period Relative Risk (RR) curves for incidence, prevalence, and mortality, respectively. Panels **(G)**, **(H)**, and **(I)** represent the Cohort RR curves for incidence, prevalence, and mortality, respectively.

To further analyze the burden of IHD, we employed an APC model (Supplementary Tables S1–S3). From the longitudinal age curve perspective, both female and male populations exhibited an overall exponential increase in disease burden with advancing age, with males consistently having a higher burden than females. A notable difference was observed in the male cohort, where the incidence rate and mortality rate began to decline within the age range of 95–100 years (Figures 3A–C).

In terms of the period effect, the incidence rate began to rise between 1992 and 1997, peaking in the period from 2007 to 2012 before showing a subsequent periodic decline (Figure 3D). The prevalence rate continued to increase throughout the entire period; however, the prevalence rate among males started to exhibit a slight decline during the 2017–2021 period, while it continued to rise among females (Figure 3E). Female mortality rates have shown a consistent decline since the 2002–2007 period, while male mortality rates began to decline after the 2012–2017 period (Figure 3F).

From the cohort perspective, both the incidence rate and prevalence rate of IHD increased progressively with the proximity of birth years (Figures 3G,H). The mortality rate for females exhibited an inverted U-shaped curve, peaking among those born between 1935 and 1940 (Figure 3I). In contrast, the trends within the male cohort appeared more complex; overall, the mortality rate increased with the advancing birth years. However, for males born between 1985 and 1990, a decreasing trend began to emerge, particularly noticeable in those born during the 2000–2005 period.

### Decomposition analysis of the burden of IHD

**Figure 4:**
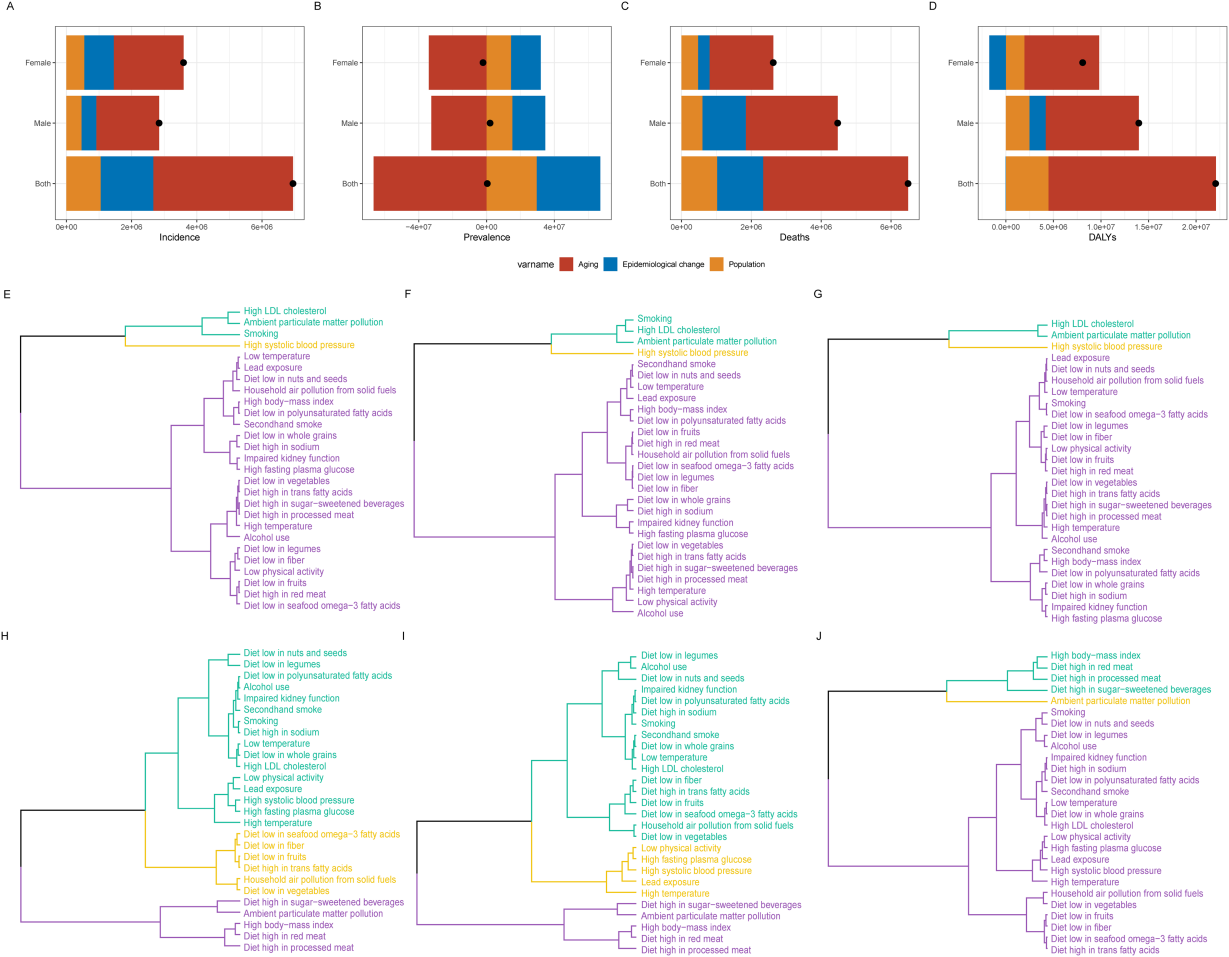
Decomposition analysis of IHD in China, 1990–2021: **(A)** prevalence, **(B)** incidence, **(C)** mortality, **(D)** disability-adjusted life years (DALYs). Black dots represent the overall changes attributed to population growth, aging, and epidemiological transitions. Clustering analysis results of 27 risk factors contributing to IHD mortality and DALYs burden in China in 2021: **(E)** Both sexes, **(F)** Males, **(G)** Females. Clustering analysis results of 27 risk factors contributing to changes in IHD mortality and DALYs burden in China from 1990 to 2021: **(H)** Both sexes, **(I)** Males, **(J)** Females.

The indicators of IHD are primarily associated with age (Figures 4A–D). In the contribution to incidence rates, age accounted for 67.74% for males and 59.64% for females (Supplementary Table S4). For mortality rates, the contributions were 58.84% for males and 69.44% for females. Interestingly, age displayed an opposing effect on prevalence rates, contributing −1585.16% for males and −1628.69% for females. Regarding DALYs, age contributed 70% for males and 97.25% for females. Notably, the effect of epidemiological change was negative in females, with a contribution of −21.49%.

### Cluster analysis of risk factors for IHD

The results from the cluster analysis of disease burden based on mortality and DALYs indicate that the primary contributor to the burden of IHD in China is high systolic blood pressure (Supplementary Table S5). This is followed by ambient particulate matter pollution, high LDL-C, and smoking. Notably, the disease burden associated with smoking is predominantly observed in the male population (Figures 4E–G).

Between 1990 and 2021, regardless of gender, the most significant increases in disease burden were attributed to ambient particulate matter pollution, a diet high in processed meat, a diet high in red meat, a diet high in sugar-sweetened beverages, and a high body mass index (Supplementary Table S6). Among these factors, the increase in ambient particulate matter pollution was particularly pronounced within the female population (Figures 4H–J).

### Frontier analysis of IHD burden

**Figure 5:**
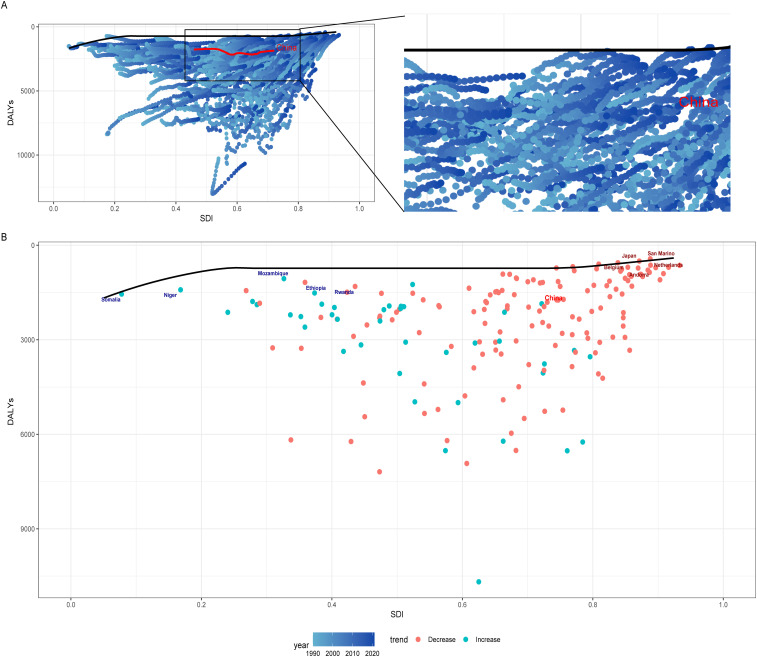
**(A)** Frontier analysis of DALYs for IHD based on the SDI from 1990 to 2021. **(B)** Frontier analysis of DALYs for IHD based on the SDI in 2021. Countries with the smallest disease burden and an SDI greater than 0.85 are marked in red, while those with the smallest disease burden and an SDI less than 0.5 are marked in blue.

The frontier analysis demonstrated the trends in ASDR from 1990 to 2021 across 204 countries in relation to the SDI (Figure 5A). Throughout this 32-year period, China exhibited marked fluctuations in ASDR for IHD in response to increasing SDI levels. The maximum values were recorded in 2005 (values = 2169.86) and 2011 (values = 2168.31), showcasing a general trend of initial increase followed by a subsequent decrease (Supplementary Table S7).

We constructed a frontier analysis chart for ASDR in 2021 across the 204 countries and identified the five countries with the smallest range of effective differences among high SDI nations (0-205.65). These countries are Japan, San Marino, Belgium, the Netherlands, and Andorra (Figure 5B).

### Forecast of burden for the next 15 years

**Figure 6:**
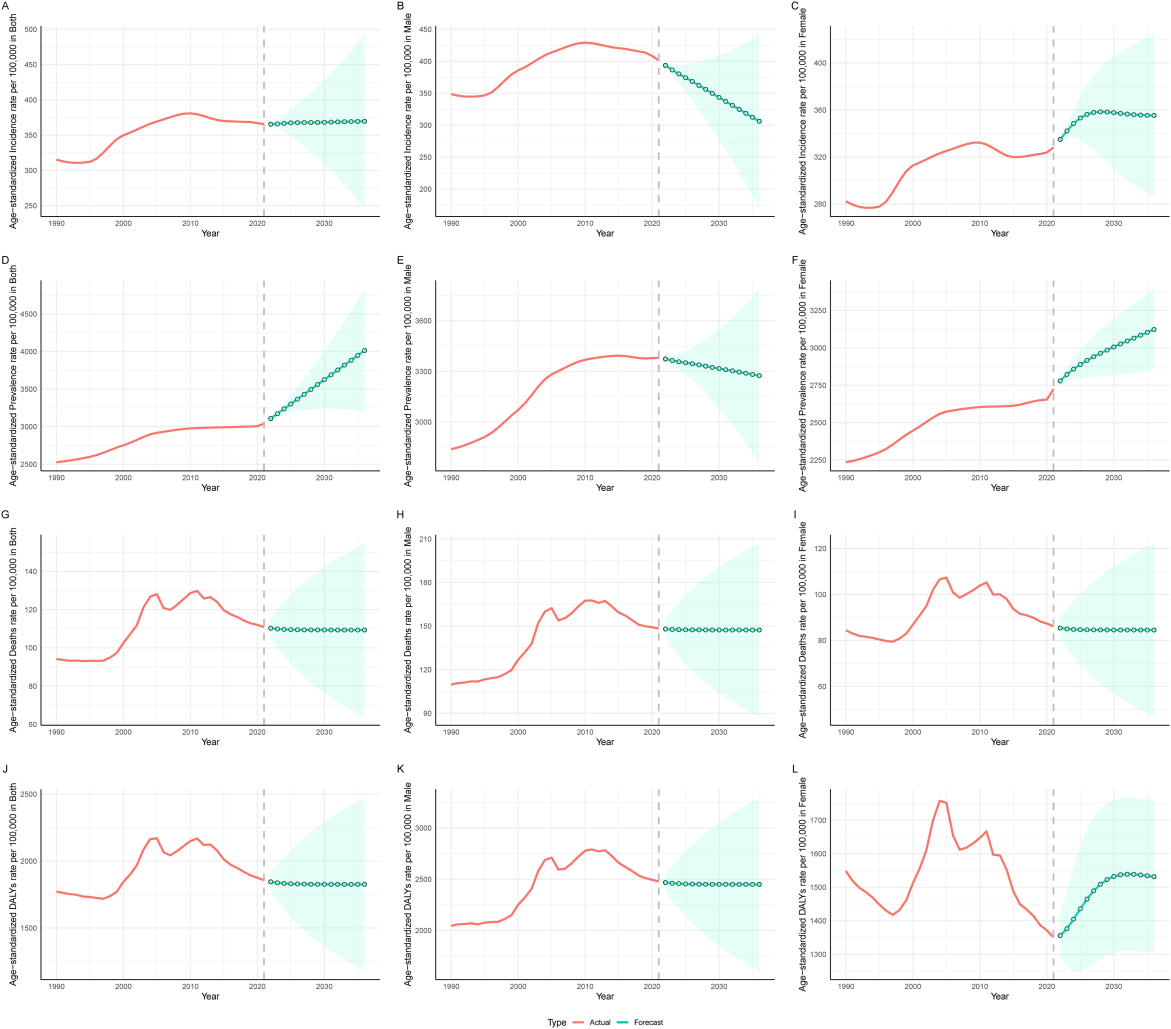
Projected trends in incidence, prevalence, mortality, and DALYs of IHD in China for the next 15 years (2022–2036). **(A**–**C)** Represent the trends in incidence for Both sexes, Males, and Females, respectively. **(D–F)** Represent the trends in prevalence for Both sexes, Males, and Females, respectively. **(G–I)** Represent the trends in mortality for Both sexes, Males, and Females, respectively. **(J–L)** Represent the trends in DALYs for Both sexes, Males, and Females, respectively. The red line indicates observed trends, while the green dashed line and shaded areas represent projected trends with 95% CI.

The ASIR of IHD in China is projected to remain relatively stable overall; however, notable differences are observed between genders (Figures 6A–C). Over the next 15 years, the ASIR for males is expected to exhibit a declining trend, decreasing from 401 cases per 100,000 in 2021 to 306 cases per 100,000 by 2036. In contrast, the ASIR for females is anticipated to rise from 328 cases per 100,000 in 2021 to 356 cases per 100,000 by 2026, after which it is expected to stabilize at this level (Supplementary Table S8).

The ASPR is projected to follow an upward trajectory, increasing from 3,042 cases per 100,000 in 2021 to 4,013 cases per 100,000 by 2035. For males, the prevalence is projected to decline mildly from 3,379 cases per 100,000 in 2021 to 3,274 cases per 100,000 by 2035, whereas for females, it is expected to rise from 2,724 cases per 100,000 to 3,122 cases during the same period (Figures 6D–F).

The ASMR is anticipated to remain relatively unchanged over the next 15 years (Figures 6G–I). Additionally, the ASDR for males are projected to remain stable, while for females, there is an expected increase from 1,350 in 2021 to 1,536 by 2031, followed by a period of stability (Figures 6J–L).

## Discussion

This study utilized the GBD database to comprehensively analyze the burden of IHD in China from 1990 to 2021. The findings revealed a substantial numerical increase in the prevalence, incidence, mortality, and DALYs associated with IHD, while ASMR and ASDR demonstrated an initial rise followed by a subsequent decline. Compared to females, males exhibited a substantially higher burden in early and middle adulthood, whereas females surpassed males in the elderly population. High systolic blood pressure, ambient particulate matter pollution, elevated LDL cholesterol, and smoking were identified as the primary contributors to the disease burden, with environmental pollution exerting a particularly pronounced impact on females. Future projections indicate that over the next 15 years, males will experience declining trends in both incidence and prevalence, while females are anticipated to exhibit increasing trends in incidence, prevalence, and DALYs.

Our study observed that the burden of IHD is generally higher in males than in females, which can largely be explained by a combination of biological and behavioral factors. One key factor is the protective role of estrogen in females, particularly before menopause. Estrogen exhibits anti-inflammatory properties, improves endothelial function, and helps regulate lipid metabolism by reducing LDL cholesterol and increasing high-density lipoprotein (HDL) cholesterol levels, thereby offering significant cardiovascular protection (16–18). In contrast, males lack this estrogen-mediated protection due to lower levels of circulating estrogen, leading to earlier and heightened exposure to atherosclerotic risk. Postmenopause, however, the risk of CVD in females increases markedly due to the decline in estrogen levels, narrowing the gender gap (19). On the other hand, females are more likely to present with atypical symptoms such as fatigue, shortness of breath, and nausea (20). These symptoms are less likely to be recognized as cardiac-related and are often misattributed to other conditions, particularly because traditional diagnostic criteria and clinical guidelines have been primarily developed based on male populations. Consequently, females may not receive timely interventions, leading to poorer outcomes (16, 21). This shift aligns with our observations of higher IHD burden in elderly females.

Behavioral factors further exacerbate the disparity. Males are more likely to engage in smoking and excessive alcohol consumption, both of which significantly elevate the risk of atherosclerosis and coronary artery disease (22, 23). Additionally, males tend to develop hypertension and diabetes—key risk factors for IHD—earlier in life compared to females (24, 25). These risk profiles contribute to the higher incidence and prevalence observed in males during middle adulthood.

We observed that the burden of IHD rose rapidly and remained at relatively high levels between 1995 and 2010, driven by multiple contributing factors. Rapid urbanization during this period led to significant lifestyle changes, including reduced physical activity, unhealthy dietary habits, and increased consumption of high-fat and high-sugar foods, all of which heightened CVD risk (26). Additionally, public awareness of cardiovascular risk factors such as hypertension, diabetes, and hyperlipidemia remained low, resulting in poor prevention and management of these conditions (27).

Since 2010, the burden of IHD has shown a gradual decline, which can be attributed to several key factors. The Chinese government has launched large-scale health campaigns aimed at raising public awareness of cardiovascular risk factors, promoting smoking cessation, and encouraging healthier lifestyles (28–30). Advances in medical technology and improved access to healthcare have facilitated early diagnosis and better management of hypertension, diabetes, and hyperlipidemia (31, 32). Additionally, increased government focus on chronic disease prevention, particularly hypertension control and blood pressure monitoring programs, has played a critical role in mitigating the burden of IHD during this period (33, 34).

Decomposition analysis indicates that aging plays a critical role in the burden of IHD. This can be attributed to age-related structural and functional changes in the vascular system, such as reduced elasticity, arterial stiffness, and endothelial dysfunction. These changes heighten the susceptibility of arteries to blockage and ischemia (35). Moreover, older adults are more likely to develop comorbidities, including chronic kidney disease, HF, and cerebrovascular disease, which further amplify their cardiovascular risk (36, 37). Additionally, with advancing age, individuals experience prolonged exposure to key cardiovascular risk factors such as hypertension, diabetes, and hyperlipidemia, further contributing to the increased burden of IHD in the elderly population.

In China, the primary contributors to deaths and DALYs caused by IHD are hypertension, ambient particulate matter pollution, high LDL cholesterol, and smoking (particularly among males). These findings are consistent with previous surveys and research (13, 38–40). Among the risk factors contributing to the increasing burden of IHD from 1990 to 2021, the most significant changes were observed in ambient particulate matter pollution, high-energy diets, and obesity. These findings underscore the critical importance of environmental protection and the adoption of healthy lifestyles. Effective policies aimed at reducing air pollution, combined with public health campaigns promoting balanced diets and regular physical activity, are essential for mitigating the IHD burden.

From the 32-year trends and future projections, the incidence rate and DALYs of IHD in the female population are expected to rise. This implies a growing number of female patients, which will result in increased healthcare demands. Greater attention should be paid to the health of the female population, emphasizing preventive measures and lifestyle modifications. Additionally, the burden of IHD remains concentrated in middle-aged men and elderly women, who should receive more focused attention and healthcare resources. This includes early detection of risk factors and timely interventions to prevent the onset and progression of the disease. Frontier analysis has identified Japan as one of the countries with the lowest IHD burden in high-SDI regions. Given the cultural similarities between Japan and China, Japan's healthcare practices and related policies provide valuable insights. As China's SDI continues to advance, adopting Japan's successful strategies—such as comprehensive preventive care programs and effective public health policies (41–43)—could help reduce the IHD burden and improve population health outcomes.

This study utilized the GBD database to conduct a detailed analysis of IHD in China, focusing on trends in incidence, prevalence, mortality, and DALYs over time, as well as age and gender characteristics and future projections. Additionally, the study identified and analyzed the major risk factors contributing to the disease burden. However, there are some limitations. The data used in this study are aggregated at the national level, which may obscure significant regional or local variations. This makes the findings less suitable for guiding individual-level research or interventions. Furthermore, the reliance on secondary data may introduce biases, such as potential inaccuracies in data collection, diagnostic errors, or reporting biases. Additionally, the GBD database aggregates a wide range of diseases into broader categories, which can obscure specific health issues that may require targeted interventions. Future studies should aim to incorporate more granular, region-specific data to better capture local patterns and inform tailored public health strategies.

## Conclusions

The burden of IHD in China has evolved significantly over the past three decades, driven by demographic and environmental factors. While prevalence and incidence have risen, mortality and DALYs have shown a recent decline, reflecting shifts in disease patterns. Age and gender disparities are evident, with middle-aged males and elderly females disproportionately affected. Key contributors, such as high blood pressure and pollution, highlight the need for targeted interventions. Gender-specific public health strategies, alongside improved environmental and health policies, are essential to mitigating the future burden of IHD in China.

## Data Availability

Publicly available datasets were analyzed in this study. This data can be found here: The Global Burden of Disease study 2021 is an open-access resource; data are available at https://vizhub.healthdata.org/gbd-results.
